# Host-Based Treatments for Severe COVID-19

**DOI:** 10.3390/cimb45040203

**Published:** 2023-04-05

**Authors:** Lucrezia Mondini, Francesco Salton, Liliana Trotta, Chiara Bozzi, Riccardo Pozzan, Mariangela Barbieri, Stefano Tavano, Selene Lerda, Michael Hughes, Marco Confalonieri, Paola Confalonieri, Barbara Ruaro

**Affiliations:** 1 Pulmonology Unit, Department of Medical Surgical and Health Sciences, University Hospital of Cattinara, University of Trieste, 34149 Trieste, Italy; lucrezia.mondini@asugi.sanita.fvg.it (L.M.); francesco.salton@gmail.com (F.S.); liliana.trotta@asugi.sanita.fvg.it (L.T.); chiara.bozzi@asugi.sanita.fvg.it (C.B.); riccardo.pozzan@asugi.sanita.fvg.it (R.P.); mariange.barbieri@gmail.com (M.B.); stefano.tavano@asugi.sanita.fvg.it (S.T.); paola.confalonieri.24@gmail.com (P.C.); barbara.ruaro@yahoo.it (B.R.); 2 Graduate School, University of Milan, 20149 Milano, Italy; selenelerda@gmail.com; 3 Division of Musculoskeletal and Dermatological Sciences, Faculty of Biology, Medicine and Health, The University of Manchester & Salford Royal NHS Foundation Trust, Manchester M6 8HD, UK; michael.hughes-6@manchester.ac.uk

**Keywords:** SARS-CoV-2, immune-system, cytokine storm, immunomodulation

## Abstract

COVID-19 has been a global health problem since 2020. There are different spectrums of manifestation of this disease, ranging from asymptomatic to extremely severe forms requiring admission to intensive care units and life-support therapies, mainly due to severe pneumonia. The progressive understanding of this disease has allowed researchers and clinicians to implement different therapeutic alternatives, depending on both the severity of clinical involvement and the causative molecular mechanism that has been progressively explored. In this review, we analysed the main therapeutic options available to date based on modulating the host inflammatory response to SARS-CoV-2 infection in patients with severe and critical illness. Although current guidelines are moving toward a personalised treatment approach titrated on the timing of presentation, disease severity, and laboratory parameters, future research is needed to identify additional biomarkers that can anticipate the disease course and guide targeted interventions on an individual basis.

## 1. Introduction

The SARS-CoV-2 coronavirus was first identified as a cause of severe pneumonia in Wuhan, but it quickly spread worldwide, and on 11 March 2020, the World Health Organization (WHO) declared COVID-19 a global pandemic [1]. Currently, more than 750 million confirmed cases of COVID-19 have been reported globally. Currently, COVID-19 infection still has a significant burden on the global health care system, and more than 6 million deaths have been reported [2].

The manifestations of SARS-CoV-2 infection range from asymptomatic or mild forms of flu-like illness to severe forms of bilateral interstitial pneumonia resulting in acute respiratory failure requiring maintenance therapies and admission to an intensive care unit.

The WHO has classified COVID-19 as mild, moderate, severe, and critical illness. Mild illness consists of symptoms of SARS-CoV-2 infection (such as fever, cough, fatigue, wheezing), but without manifestations of viral pneumonia or hypoxia. Moderate, severe, and critical illness share common evidence of pneumonia, with an increasing severity of lung involvement and then progressively worsening clinical manifestations. Patients with moderate COVID-19 are defined by clinical signs of pneumonia with SpO2 ≥ 90% with room air. Critical illness consists of clinical signs of pneumonia with at least one of the following: respiratory rate > 30 breaths/min, severe respiratory distress, or SpO2 < 90% in room air. Critical illness is identified by Acute Respiratory Distress Syndrome (ARDS, according to the 2012 Berlin definition [3]), sepsis, septic shock, or any other condition requiring life-sustaining therapies [4,5].

The scientific community has produced a vast number of studies to identify the pathogenetic mechanisms of this disease and develop effective therapeutic strategies.

One of the mechanisms by which SARS-CoV-2 enters cells is through ACE2 receptors, which are ubiquitously expressed in several organs, including the lungs, gastrointestinal tract, kidneys, heart, and nervous system [6,7].

The hyperactivation of innate immunity signalling pathways following SARS-CoV-2 infection can lead to massive systemic inflammation, also known as a cytokine storm, which is the main cause of multi-organ damage and death [8].

In fact, patients with severe COVID-19 disease show persistently elevated blood levels of interferon 𝛾 (IFN-𝛾), interleukin-1 (IL-1), interleukin-6 (IL-6), interleukin-12 (IL-12), transforming growth factor 𝛽 (TGF-𝛽), monocyte chemoattractant protein-1 (MCP-1), and interleukin-8 (IL-8) [9], involving both systemic and microvascular circulation, documented by nailfold capillaroscopy [10,11,12].

Based on the concept that the immune response can be modulated, several therapeutic approaches have been developed for the treatment of severe COVID-19. In addition, recent evidence has shown that immuno-profiling can guide therapeutic choices with newly available target therapies in patients with severe COVID-19 [13,14]. In this review, we analyse the most relevant treatments for severe COVID-19 among those that have been shown to be effective and are recommended by current guidelines, with a focus on molecular and clinical features that may serve for a more effective host-based therapeutic approach, as listed in Table 1. Specifically, this critical review considers studies on therapeutic approaches for which one or more randomised clinical trials have been conducted compared with placebo with at least one positive result in terms of achieving the primary outcome.

## 2. Corticosteroids

Corticosteroids are recommended by the current WHO guidelines as first-line therapy in hospitalised patients with severe and critical management of COVID-19 [4,15].

Glucocorticoids (GCs) are endogenous modulators of systemic inflammation in addition to exerting a number of other genomic and nongenomic effects. Indeed, cytokines secreted during the innate immune response (e.g., IL1-b and IL-6) activate the hypothalamic–pituitary–adrenal axis to secrete GCs, which exert their immunomodulatory function by binding the ubiquitously expressed glucocorticoid receptor-α (GR-α). When there is a massive inflammatory response, such as in severe COVID-19, glucocorticoid-mediated anti-inflammatory activity may be inadequate for the severity of the patient’s critical illness (corticosteroid-related critical illness failure, CIRCI) [16,17].

Glucocorticoids had already been shown to be effective in reducing mortality in sepsis, severe pneumonia, or ARDS from any cause.

Several studies have examined the use of GCs in community-acquired pneumonia (CAP) on the assumption that the immunomodulating effect of these drugs was able to control inflammation and thus promote the healing process and avoid complications, particularly those related to sepsis, by reducing lung and systemic inflammation, especially in severe CAP [18,19]. The extensive literature related to this therapeutic approach has been analysed over time in several reviews and meta-analyses that have confirmed a more rapid improvement in the clinical parameters of severe forms of CAP treated with GCs, but without always reflecting a decrease in mortality or ICU stays [20,21]; in fact, the American Thoracic Society (ATS) guidelines emphasise that the use of GCs in severe forms of CAP is still partially controversial and requires further research in this area [22]. In order to elucidate a reasoned use of steroid therapy in patients with ARDS, in 2020, Meduri et al. reviewed the pharmacological principles underlying the use of GCs, which should guide both the timing of the use of these drugs and their dosing and tapering; they also suggested the clinical and laboratory parameters that should be monitored during therapy, highlighting how steroid therapy should be individualised for each patient in order to have a successful outcome [23].

Based on these assumptions and a multicentre, retrospective cohort study by the Saudi Critical Care Trial Group that advised against the use of GCs in critically ill patients with Middle East Respiratory Syndrome (MERS) [24], the WHO initially did not recommend the use of steroid therapy for the treatment of SARS-CoV-2 infections [25]. 

Despite initial scepticism about the use of GCs in COVID-19 disease, the extensive literature reviewed in this critical review subsequently validated the immunomodulatory effect of these drugs and confirmed the rationale for their use in routine clinical practice.

In June 2020, the RECOVERY clinical trial showed for the first time, in a prospective randomised trial compared with placebo, that treatment with 6 mg dexamethasone for up to 10 days reduced the risk of death at 28 days in patients hospitalised with COVID-19 undergoing mechanical ventilation or oxygen therapy, with a greater impact among patients hospitalised with COVID-19 receiving respiratory support with mechanical ventilation (36% reduction) compared with oxygen therapy (18% reduction). In contrast, GCs showed an increased risk of death among patients who did not require respiratory support at the time of randomisation [26].

Since the RECOVERY trial, subsequent clinical trials have focused on patients with severe COVID-19.

The multicentre, randomised, open-label, CODEX clinical trial, conducted in 41 intensive care units (ICUs), tested the efficacy of intravenous dexamethasone plus standard of care versus placebo on ventilator-free and ventilator-free days in patients with moderate and severe ARDS due to SARS-CoV-2 infection. CODEX confirmed a statistically significant increase in the number of ventilator-free days over 28 days in patients treated with dexamethasone (6.6 ventilator-free days versus 4.0 in the standard care group) [27].

The COVID-STEROID 2 study group proposed treatment with higher doses of dexamethasone. This multicentre clinical trial randomised 1,000 patients with SARS-CoV-2 pneumonia and severe hypoxemia in a 1:1 ratio to receive either 12 or 6 mg of intravenous dexamethasone for up to 10 days and found no statistically significant differences by treating patients with a higher dose of steroids. In fact, the two groups showed a similar number of days of life without life support at 28 days [28].

The REMAP-CAP study proposed the use of an alternative steroid molecule to dexamethasone: 384 patients with confirmed SARS-CoV-2 infection admitted to the ICU were randomised to receive a fixed dose of hydrocortisone (50 or 100 mg intravenously) or a shock-dependent dose of hydrocortisone compared with standard of care alone. 

This study confirmed the superiority of steroid therapy with hydrocortisone over the 2020 standard of care, showing an improvement in organ support-free days within 21 days [29].

Dequin et al. also analysed the effect of intravenous hydrocortisone for 7 days at higher doses (200 mg) in a multicentre randomised double-blind sequential trial (CAPE COVID) involving 149 critically ill patients with SARS-CoV-2 pneumonia. This study was stopped early but showed no improvement in organ support-free days within 21 days and showed no statistically significant differences between the placebo and steroid groups in treatment failure (defined as death or persistent respiratory support) at day 21 [30].

Finally, several clinical trials have proposed a treatment protocol with methylprednisolone. 

Edalatifard et al. proposed three-day treatment with pulsed intravenous methylprednisolone (250 mg daily) versus placebo in a randomised controlled, single-blind, two-arm, parallel clinical trial. This study included 68 patients randomised in a 1:1 ratio with COVID-19 infection, abnormal computed tomography (CT) findings, and oxygen saturation (SpO2) < 90% at rest. Patients treated with methylprednisolone showed a lower mortality rate than the standard of care (5.9% vs. 42.9%). In addition, patients in the methylprednisolone group who required mechanical ventilation or high-flow oxygen (HFO) or low-dose oxygen via nasal canula were characterised by a lower incidence of death (7.7%, 8.3%, and 0%, respectively, compared with 60%, 57.1%, and 22% in the standard group) [31].

Metcovid was a parallel, double-blind, placebo-controlled, randomised, phase IIb clinical trial that proposed a lower dose of intravenous methylprednisolone (0.5 mg/kg) for a longer period (5 days) and included a larger cohort of patients. A total of 416 patients were randomised to receive methylprednisolone versus standard of care in a 1:1 ratio. The inclusion criteria included clinical and radiological signs of COVID-19, SpO2 ≤ 94% in room air, or the need for oxygen support (either low-dose or high-dose oxygen, invasive or non-invasive mechanical ventilation). Metcovid showed no statistically significant differences in 28-day mortality between the two groups, suggesting that 5 days of low-dose methylprednisolone did not change the patients’ prognosis [32].

In a multicentre, observational, longitudinal study, Salton et al. evaluated the efficacy of a protocol of infusion of 80 mg methylprednisolone daily for at least 14 days and until clinical improvement [33] in 83 hospitalised patients with confirmed SARS-CoV-2-related ARDS, PaO2/FiO2 < 250 mmHg, and CRP > 100 mg/L compared with 90 unexposed controls who met the same inclusion criteria. In this study, 28-day mortality was significantly reduced in the methylprednisolone-treated group (adjusted HR, 0.29) along with increased days free of mechanical ventilation and shorter median duration of invasive mechanical ventilation among patients admitted to the ICU [34]. Despite the methodological limitations arising from the observational design, this study was the first to investigate a titration of GC treatment duration on clinical status, assessed by a reduction in CRP and an increase in PaO2/FiO2. This is in agreement with the previous 2017 task force proposal of the Society of Critical Care Medicine and the European Society of Intensive Care Medicine for the management of critically ill patients with sepsis and septic shock, acute respiratory distress syndrome, and major trauma [35]. 

Overall, the REACT working group published a meta-analysis of seven clinical trials that confirmed the reduction in 28-day mortality with steroid use in patients with COVID-19 severe pneumonia. The clinical trials analysed by the REACT study examined different molecules: low-dose and high-dose dexamethasone, low-dose hydrocortisone, and high-dose methylprednisolone [36].

Thus, once corticosteroid therapy for severe COVID infections was validated, the clinicians focused on exploring the most appropriate molecules, dosage, and timing of administration. 

Ranjbar et al. compared dexamethasone and methylprednisolone in a prospective randomised, triple-blind controlled trial, assigning 86 hospitalised COVID-19s into two groups treated with higher doses of methylprednisolone (2 mg/kg/day) or dexamethasone (6 mg/day) for 10 days. Overall, the group treated with methylprednisolone showed better results than the group treated with dexamethasone. In particular, patients treated with methylprednisolone had significantly better clinical status at days 5 and 10. In addition, treatment with methylprednisolone resulted in a shorter length of hospital stay and less need for ventilatory support (18.2% compared with 38.1% in the dexamethasone group) [37].

The same drug protocols were analysed by Saeed et al. in a prospective cohort study of 414 COVID-19 patients with ARDS, according to Berlin definition [3], and who were mechanically ventilated. Patients treated with methylprednisolone showed a significant reduction in ICU length of stay (7.33 in the methylprednisolone group vs. 19.43 + 4.42 in the dexamethasone group). This study also analysed the effect of the two different pharmacological protocols on biological markers: the two groups of patients showed similar blood levels of C-reactive protein (CRP), D-dimer, cytokines, and LDH at the time of ICU admission, while the same inflammatory markers were significantly lower in the methylprednisolone group after 10 days [38].

These studies seem to suggest the use of methylprednisolone in subgroups of patients with severe SARS-CoV-2 pneumonia; however, further analysis of larger samples is needed to generalise these findings.

The Italian randomised controlled open-label clinical trial (MEDEAS) examined a protocol of infusional methylprednisolone at an initial dose of 80 mg/day for 7 days, and then gradually reduced every 3 days only if the patient had a PaO2:FiO2 > 200 compared with dexamethasone 6 mg/day for 10 days or until hospital discharge (whichever was earlier) in 677 participants, randomised into two parallel arms with a 1:1 allocation ratio, showing no statistically significant differences in 28-day mortality in the two arms. Despite the non-superiority of one drug over the other, MEDEAS also showed that the arm of patients treated with methylprednisolone was characterised by longer mean hospitalisation times, partly because of the inherent duration of the treatment itself, but by a lower incidence of intensive care unit admissions. This is, to date, the only multicentre, open-label, randomised controlled clinical trial comparing the two treatment protocols and which considered the largest number of patients, involving 26 centres throughout Italy [39]. In conclusion, the current WHO guidelines suggest the use of any GC molecule, at a dose equivalent to 6 mg dexamethasone for 7–10 days, in moderate and severe COVID-19 [4]. This treatment regimen has been shown to be safe and effective in several randomised clinical trials, reducing all-cause mortality without being associated with a higher incidence of complications or adverse events than usual care. Despite its unquestionable efficacy, it is likely that this protocol could be further improved. Indeed, it is still unclear which GCs molecule is the best between dexamethasone and methylprednisolone, and more importantly, future research should be directed at investigating possible biomarkers of the hyperinflammatory state that could serve as a guide to modulate the dose and duration of GC therapy on an individual basis. In this regard, there is still uncertainty as to why even younger patients without comorbidities sometimes have a more severe course of both SARS-CoV-2-related and unrelated pneumonia. One possible explanation stems from the high variability in the plasma GC levels following administration of the same exogenous dose, which may be related to some degree of glucocorticoid resistance observed in some individuals in terms of CRP reduction and time to clinical improvement [16]. Consequently, it is likely that the GC dose should be titrated according to the level of GC resistance. Some cytokines have been proposed as possible biomarkers of GC resistance, e.g., IL-8, but their role and possible clinical application require further studies in larger populations [14].

## 3. Tyrosine Kinase Inhibitor

Janus kinase/signal transducer and activator of transcription proteins (JAK/STAT) is the key signalling pathway shared by the various inflammatory mediators involved in cytokine storms (especially IL-6, INF--γ, (G-CSF), IL-2, and others), resulting in the attraction of macrophages and neutrophils responsible for tissue damage [40].

Based on this notion, treatments with JAK/STAT inhibitors, such as baricitinib or ruxolitinib, selected JAK1/JAK2 inhibitors, have been proposed in COVID pneumonia [41]. In May 2022, the FDA approved the use of baricitinib in hospitalised adults with COVID-19 infection requiring oxygen supplementation, non-invasive mechanical ventilation, or ECMO. This position, derived from previous evidence, has been shared by all major guidelines for the management of severe COVID-19 [42,43].

The first study, called COV-BARRIER, was conducted in 2021 as a randomised, double-blind, parallel-group, placebo-controlled trial in which hospitalised patients with evidence of SARS-CoV-2 pneumonia were randomly assigned to receive baricitinib or placebo, in addition to standard of care. The study showed a reduction in 28- and 60-day mortality in patients hospitalised in the baricitinib plus standard of care arm; specifically, 39% of patients died in the baricitinib group compared to 58% on placebo—a 19% absolute risk reduction.

The authors pointed out that COV-BARRIER was an initial exploratory study, so they conducted a phase 3 study to further investigate the results, studying the effect of baricitinib in critically ill patients requiring invasive mechanical ventilation (IMV) or extracorporeal membrane oxygenation (ECMO). The results of this phase 3 study confirmed a reduction in all-cause mortality at 28 and 60 days (38.2%) in the baricitinib group compared with placebo [44].

Confirming previous results, the ACTT-1 group conducted a randomised, double-blind, placebo-controlled trial evaluating remdesivir alone versus remdesivir plus baricitinib in a group of 1033 patients hospitalised with COVID-19 pneumonia, including the subgroup receiving high-flow oxygen (HFO) or non-invasive ventilation (NIV). This study demonstrated reduced recovery time and rapid improvement in clinical status in the baricitinib plus remdesivir arm, especially in patients who required HFO or NIV [45].

Bronte et al. also examined the effect of baricitinib on serum cytokine levels. This was a longitudinal observational study in which 20 patients with SARS-CoV-2 pneumonia were treated with baricitinib plus standard care, showing a decrease in serum levels of IL-6, IL-1β, and TNF-α and an increase in lymphocyte cells, as well as a reduction in the need for oxygen therapy with a progressive increase in the P/F ratio. This study dates back to the early stages of the pandemic, and it is important to mention that none of the included patients received systemic steroid therapy [46].

Finally, the RECOVERY study group enrolled 10,852 inpatients, 8156 of whom received standard care with baricitinib or alone. This is the largest randomised trial that tested the effect of Jak INH in patients hospitalised with SARS-CoV-2 infection and showed a significant reduction in 28-day mortality in the baricitinib lineage (12% in the baricitinib group vs. 14% in the usual care group). However, RECOVERY also shows that the benefit of baricitinib therapy was less than suggested by previous studies [47].

Cao et al., conducted a multicentre, single-blind, randomised, controlled trial of 43 hospitalised patients randomly assigned to receive ruxolitinib or placebo both with standard of care and showed no faster clinical improvement in patients who received ruxolitinib, but lower levels of cytokines in the blood (IL-6, nerve growth factor b, IL-12 (p40), macrophage migration inhibitory factor, MIP-1a, macrophage inflammatory protein 1b (MIP1b), and vascular endothelial growth factor (VEGF)) in the ruxolitinib group [48].

These data were confirmed by the randomised, double-blind, placebo-controlled, phase 3 RUXCOVID trial, which demonstrated no benefit in treatment with ruxolitinib in 432 patients hospitalised with SARS-CoV-2 pneumonia who required oxygen supplementation, randomly assigned to receive ruxolitinib or placebo with the standard of care [49].

Currently, RCTs in hospitalised patients with SARS-CoV-2 pneumonia are testing Bruton’s tyrosine kinase inhibitors (ibrutinib, acalabrutinib, and zanubrutinib), phosphatidylinositol-3-kinase (PI3K)/mammalian target of rapamycin (mTOR) inhibitors (duvelisib or temsirolimus), and other JAK inhibitors (tofacitinib) [50].

The studies available to date have therefore endorsed the use of baricitinib, with the FDA’s current indications approving the use of this drug for adult hospitalised patients treated with high-dose oxygen, NIV, or ECMO and systemic steroids. In contrast, however, the EMA has not yet approved the use of this drug in Europe. The above-mentioned RCTs currently ongoing may, in the future, offer therapeutic alternatives to the use of baricitinib [50,51]. 

## 4. Anti-Cytokine Treatment

IL-1 and IL-6 play key roles as mediators of inflammation secondary to SARS-CoV-2 infection. The dysregulation and overactivation of signalling pathways mediated by these cytokines are implicated in the development of severe COVID-related symptoms [52]. 

Specifically, dendritic cells and mononuclear macrophages, activated by PPRs (pattern recognition receptors), secrete IL-1 and IL-6, which maintain and sustain the acute inflammatory reaction through the activation of innate immunity. IL-6 also interacts with T cells and is responsible for dysregulated T-cell activation in SARS-CoV-2 disease [40].

Based on these reasons, a number of treatments with anti-IL-6 (tocilizumab, sarilumab) or anti-IL-6 (siltuximab) and anti-IL-1 (anakinra) monoclonal antibodies have been proposed [53,54]. 

In light of the scientific evidence from the various clinical trials, in June 2021, the US Food and Drug Administration (FDA) cleared tocilizumab in hospitalised patients (both adults and paediatric) receiving systemic steroids and requiring supplemental oxygen, NIV, or ECMO.

The RECOVERY group enrolled 4116 COVID-19 patients with hypoxia and increased C-reactive protein (CRP) who were randomised to receive tocilizumab or placebo plus standard of care and found that tocilizumab reduced 28-day mortality (31% of patients receiving tocilizumab died at 28 days vs. 38% of patients treated with standard of care) and progression to NIV (35% vs. 42% respectively; hazard ratio 0.84) [55].

At the same time, Rosas et al. enrolled 452 patients with severe COVID-19 pneumonia to receive tocilizumab versus placebo in a 2:1 ratio (COVACTA study) and found no significant differences in mortality between the two groups, but the secondary outcome showed a reduction in ICU and hospital discharge times [56]. The EMPACTA trial of 389 patients hospitalised for severe COVID-19 pneumonia who did not require NIV, randomised to receive tocilizumab or placebo, confirmed that tocilizumab therapy did not change mortality, but reduced the risk of worsening respiratory function and the need for NIV [57].

Somers et al. evaluated the efficacy of tocilizumab with an observational, controlled study focusing on a subgroup of critically ill, mechanically ventilated patients with SARS-CoV-2 pneumonia. This study confirmed a reduction in the mortality in patients treated with tocilizumab. In addition, the incidence of secondary bacterial nosocomial pneumonia was shown to be increased in these patients, but did not affect the final outcome [58].

The REMAP-CAP research group compared tocilizumab or sarilumab versus placebo in 803 critically ill COVID-19 patients in the ICU receiving organ support, enrolled within 24 h. This study confirmed a reduction in mortality among patients treated with IL-6 receptor blockade compared with controls (27% vs. 36%, respectively), associated with a shorter recovery time. In addition, the REMAP-CAP emphasised the importance of identifying patient characteristics that may predict better response to IL-6R inhibitor drugs, such as blood levels of RCP and IL-6, and the importance of early treatment in patients at higher risk of SARS-CoV-2 disease progression [59]. 

The evidence on the use of sarilumab is still not unambiguous; for example, an Italian open-label study of 56 patients with severe COVID-19 pneumonia resulting in respiratory failure and elevated blood levels of IL-6 found no reduction in mortality in the group of 28 patients treated with sarilumab [60].

Confirming the previous findings, the Sarilumab COVID-19 Global Study Group conducted a multinational, phase 3, randomised, double-blind, placebo-controlled, 60-day study involving 420 patients with COVID-19 who required supplemental oxygen, randomly assigned to receive 200 or 400 mg of sarilumab versus placebo. In this cohort of patients, there was no reduction in mortality following treatment with sarilumab, but it should be considered that a wide range of patients with different baseline characteristics and, thus, wide variability in oxygen requirements and blood levels of IL-6 or CRP were examined. It should be noted that in patients who required non-invasive or invasive ventilation or ECMO, a 9% higher 29-day survival rate was found in the sarilumab-treated group compared with placebo, which might suggest that treatment with this monoclonal antibody becomes meaningful in more severe patients [61].

Regarding siltuximab, at least four clinical trials are currently underway to determine the possible efficacy of this drug in patients with critical illness. In particular, an Italian observational study (SYLVANT) recently completed the enrolment phase of patients with ARDS secondary to COVID-19 pneumonia treated with siltuximab. 

The SAVE-MORE trial is a double-blind randomised controlled trial that proposed the use of anakinra in patients with rapidly deteriorating respiratory function. It also proposed a set of parameters that can enable physicians to quickly identify this type of patient so that effective treatment can be set up as soon as possible. Specifically, SAVE-MORE proposed using the level of soluble urokinase plasminogen activator receptor (suPAR) in the blood as an index of the rapid deterioration of respiratory function, and based on the values found, it proposes treatment with anakinra. The importance of this study lies in the fact that it emphasises how early treatment, which anticipates the damage induced by the inflammatory hyperactivation typical of severe forms of SARS-CoV-2, is able to change the course of the disease by significantly reducing the risk of worse clinical outcomes at day 28 [62].

Huet et al. explored the use of anakinra in patients hospitalised with severe SARS-CoV-2 through the prospective Ana-COVID cohort study, which included 99 patients, 52 of whom were treated with anakinra characterised by a reduction in the need for invasive mechanical ventilation in the ICU and mortality [63]. 

Finally, a systematic review and patient-level meta-analysis by the International Collaborative Group for Anakinra in COVID-19 analysed the vast body of available literature and confirmed that anakinra could be useful in patients with COVID-19, especially in those who show signs of hyperinflammation, e.g., elevated blood PCR values [64].

On the basis of this amount of data, tocilizumab has been confirmed for use in clinical practice to treat severe SARS-CoV-2 pneumonia; anakinra is also suggested for the treatment of COVID-19 infections resulting in the need for supplementary oxygen, with this drug being particularly useful in preventing progression to forms of severe respiratory failure. The data available so far, however, have emphasised that anakinra is only effective, and therefore indicated, if the treated patients have high blood levels of suPAR [4,50,51]. 

## 5. Anti-Complement Therapies: Anti-C5a

The innate immune response to the pathogen leads to the activation of complement pathways (lectin, classical, or alternative pathways), particularly after SARS-CoV-2 infection. The key complement components C3 and C5 are cleaved by C3 and C5 convertases into active C3a and C3b, and C5a and C5b, respectively. C3a and C5b are active anaphylatoxins that mediate the inflammatory response [65]. C5a, in particular, plays a key role in maintaining inflammation by recruiting and activating neutrophils and monocytes. Accordingly, several studies have suggested the use of drugs that can block chain events related to complement activation to modulate the inflammatory response [66,67].

In addition, elevated serum levels of C5a have been found in patients with severe SARS-CoV-2 pneumonia [68].

The PANAMO clinical trial is an exploratory, open-label, randomised trial that tested the benefit and safety of vilobelimab, an IFX-1 monoclonal antibody, a selective blocker of C5a. Initially, a phase 2 study enrolled 30 patients with severe COVID-19 pneumonia, documented by radiologic findings of pulmonary infiltrates, PaO2/FiO2 between 100 mm Hg and 250 mm Hg, or need for invasive or non-invasive ventilation, to be randomly assigned to receive standard therapy alone or more vilobelimab. This study demonstrated the benefits and safety of vilobelimab [69]. Thus, in 2022, the PANAMO trial initiated a multicentre, phase 3, randomised, double-blind, placebo-controlled study that included 368 patients with severe COVID-19 pneumonia undergoing invasive mechanical ventilation, with PaO2/FiO2 ratios of 60–200 mm Hg, who received vilobelimab or placebo. This study showed a reduction in mortality at 28 days (32% in the vilobelimab arm versus 42% in the placebo arm) and at 60 days (35% versus 46%, respectively) in patients receiving vilobelimab, suggesting the use of these drugs as adjunctive therapy in critically ill inpatients [70].

Several clinical trials testing the efficacy of different drugs that block C5 activity are currently underway, notably a Belgian clinical trial testing Zilucoplan^®^ vs. placebo (NCT04382755) and a French trial testing avdoralimab has completed recruitment (NCT04371367).

Zilucoplan^®^ and avdoralimab are drugs recently approved for use in clinical practice and therefore were tested later than vilobelimab in SARS-CoV-2 pneumonia. Ongoing studies could provide additional data for the use of these treatments.

## 6. Interferon

After viral infection, the innate immune system responds with interferons (IFNs) to limit the severity of inflammation due to its immunomodulatory and antiviral effects. 

In 2020, the COVID-19 study group on inhaled interferon beta proposed the use of nebulised interferon beta-1a as adjunctive therapy in hospitalised patients with SARS-CoV-2 infection in a phase 2, randomised, double-blind, placebo-controlled pilot study. This study included 101 patients with mild, moderate, and severe disease and showed an overall higher probability of improvement and faster recovery from SARS-CoV-2 infection in patients receiving nebulised IFN 𝛽-1a [71].

The ACTT-3 study group conducted a double-blind, randomised, placebo-controlled trial of 969 patients with COVID-19 pneumonia who had radiographic infiltrates on imaging, SpO2 with room air of 94% or less, or who required supplemental oxygen. Treatment with remdesivir alone versus remdesivir plus interferon beta-1a administered subcutaneously was proposed. This study showed no benefit in the remdesivir plus IFN 𝛽-1a branch; moreover, patients who required high-flow oxygen showed a worse outcome when treated with IFN [72].

Several ongoing clinical trials are currently testing the efficacy of inhaled IFN, particularly in hospitalised patients with COVID-19 requiring oxygen therapy in the COV-NI trial (NCT04469491) and in children (NCT05381363).

The current data therefore do not support the use of interferon in COVID-19 pneumonia; ongoing studies could probably identify specific populations that may benefit from interferon therapy.

## 7. Anti-GM-CSF

Granulocyte–macrophage colony-stimulating factor (GM-CSF) is a cytokine released by macrophages, lymphocytes, endothelial cells, fibroblasts, and alveolar epithelial cells with immunomodulatory and pro-inflammatory effects. In the lungs, GM-CSF binds GM-CSF receptors located on type II alveolar epithelial cells and macrophages, activating their immune function and supporting surfactant homeostasis [73]. High blood levels of GM-CSF have been found in patients with severe COVID-19 disease. Furthermore, elevated levels of this cytokine appear to be specific for SARS-CoV-2 disease and have not been detected in other viral lung infections, such as influenza. In addition, the measurement of serum levels of IL-6 and GM-CSF has been proposed for the stratification of the severity of COVID-19 infection [74]. 

Indeed, there are several monoclonal antibodies that can block the interaction between GM-CSF and its receptor, such as sargramostim, lenzilumab, namilumab, otilimab, and gimsilumab [75,76,77]. 

The SARPAC clinical trial is a Belgian prospective, randomised, open-label, interventional study investigating the efficacy of sargramostim in patients with acute COVID-19 disease resulting in hypoxic acute respiratory failure, currently under publication (NCT04326920). Bosteels et al. proposed inhaled therapy with sargramostim 125 mcg twice daily for 5 days plus standard of care compared with placebo. In addition, patients who developed ARDS and required mechanical ventilation within the 5-day period switched to sargramostim 125 mcg/m^2^ body surface area intravenously once daily until day 5 [78].

There are currently several clinical trials testing the efficacy of intravenous sargramostim in patients with hypoxemia-associated COVID-19 disease (NCT04411680) and inhaled sargramostim to prevent ARDS (NCT04569877) or in mild to moderate SARS-CoV-2 disease to prevent progression to severe forms (NCT04707664; NCT04642950). 

Temesgen et al. conducted a case-cohort study of 39 patients hospitalised with COVID-19 pneumonia and a risk factor for disease progression: 12 patients were treated with three doses of lenzilumab 600 mg intravenously, while 27 patients constituted the control group and were only treated with standard care. The lenzilumab group showed faster improvement and greater reduction of inflammatory markers in the blood [77].

Current guidelines do not yet suggest treatment with GM-CSF inhibitors, as there are insufficient data available in the literature to guarantee their efficacy [79].

**Table 1:** Table of studies investigating different types of host-based treatment in different sub-groups of patients with SARS-CoV-2 infection.

Authors	Title of Article	Design	Drugs	Examined Patients	Results
Corticosteroids					
The RECOVERY Collaborative Group (2021) [26]	Dexamethasone in hospitalized patients with COVID-19	Prospective randomised trial compared with placebo	Dexamethasone 6 mg vs. placebo	2104 treated vs. 4321 placebo patients hospitalised with confirmed COVID-19	Lower mortality only in patients needing oxygen support
Tomazini BM, Maia IS, Cavalcanti AB, Berwanger O, Rosa RG, Veiga VC, et al. (2020) [27]	Effect of dexamethasone on days alive and ventilator-free in patients with moderate or severe acute respiratory distress syndrome and COVID-19: The CoDEX randomized clinical trial	Multicentre, randomised, open-label, clinical trial	Dexamethasone 20 mg IV for 5 days, 10 mg for 5 days or until ICU discharge, plus standard care or standard care alone	151 treated vs. 148 placebo patients with confirmed COVID-19 and moderate to severe ARDS	Patients with COVID-19 and moderate or severe ARDS treated with dexamethasone IV plus standard showed statistically significant increase in the number of ventilator-free days over 28 days
The COVID STEROID 2 Trial Group (2021) [28]	Effect of 12 mg vs. 6 mg of dexamethasone on the number of days alive without life support in adults with COVID-19 and severe hypoxemia: The COVID STEROID 2 randomized trial	Multicentre, randomised clinical trial	Dexamethasone 12 mg IV vs. Dexamethasone 6 mg IV	982 adults with confirmed COVID-19 requiring at least 10 L/min of oxygen or mechanical ventilation	No statistical significance on ventilator-free days over 28 days
The Writing Committee for the REMAP-CAP Investigators, Angus DC, Derde L, Al-Beidh F, Annane D, Arabi Y, et al. (2020) [29]	Effect of hydrocortisone on mortality and organ support in patients with severe COVID-19: The REMAP-CAP COVID-19 corticosteroid domain randomized clinical trial	Bayesian randomised clinical trial	Hydrocortisone (50 mg or 100 mg every 6 h or shock-dependent dosage) vs. no hydrocortisone	295 treated vs. 108 non-treated adults with severe SARS-CoV-2 pneumonia in ICU	Improvement in organ support-free days within 21 days in patients treated with hydrocortisone
Dequin P-F, Heming N, Meziani F, Plantefève G, Voiriot G, Badié J, et al. (2020) [30]	Effect of hydrocortisone on 21-day mortality or respiratory support among critically ill patients with COVID-19: A randomized clinical trial	Multicentre randomised double-blind sequential trial	Hydrocortisone 200 mg/die tapered to 100 mg and then 50 mg vs. placebo	76 treated vs. 73 placebo admitted to ICU with ARDS due to COVID-19 infection	No significant reduction in treatment failure in hydrocortisone group The study was stopped early
Edalatifard M, Akhtari M, Salehi M, Naderi Z, Jamshidi A, Mostafaei S, et al. (2020) [31]	Intravenous methylprednisolone pulse as a treatment for hospitalised severe COVID-19 patients: Results from a randomised controlled clinical trial	Single-blind, randomised controlled clinical trial	Standard care plus methylprednisolone pulse 250 mg IV vs. standard care alone	34 treated vs. 34 placebo severe hospitalised patients with confirmed COVID-19 at the early pulmonary phase	Methylprednisolone in early phase improved pulmonary involvement, oxygen saturation, dyspnoea, heart rate, respiratory rate, temperature and inflammatory markers
Jeronimo CMP, Farias MEL, Val FFA, et al. (2021) [32]	Methylprednisolone as adjunctive therapy for patients hospitalized with coronavirus disease 2019 (COVID-19; Metcovid): A randomized, double-blind, phase IIb, placebo-controlled trial	Randomised, double-blind, phase IIb, placebo-controlled trial	Methylprednisolone 0.5 mg/Kg IV vs. placebo	208 treated vs. 208 placebo hospitalised patients with COVID-19	Methylprednisolone did not reduce mortality in the overall population
Salton F, Confalonieri P, Meduri GU, Santus P, Harari S, Scala R, et al. (2020) [34]	Prolonged low-dose methylprednisolone in patients with severe COVID-19 pneumonia	Multicentre, observational, longitudinal study	Methylprednisolone 80 mg IV, followed by an infusion of 80 mg/d in 240 mL of normal saline at 10 mL/h for at least 8 days, until achieving either a PaO2:FiO2 > 350 mmHg or a CRP < 20 mg/L; then MP 16 mg o.so or 20 mg IV twice daily until CRP reached < 20% of the normal range or a PaO2:FiO2 > 400 (alternative SatO2 ≥ 95% in room air)	83 patients treated vs. 90 patients in control group with severe COVID-19 pneumonia	Early administration of prolonged, low dose MP treatment was associated with a significantly lower hazard of death and decreased ventilator dependence
Ranjbar K, Moghadami M, Mirahmadizadeh A, Fallahi MJ, Khaloo V, Shahriarirad R, et al. (2021) [37]	Methylprednisolone or dexamethasone, which one is superior corticosteroid in the treatment of hospitalized COVID-19 patients: A triple-blinded randomized controlled trial	Prospective triple-blinded randomised controlled trial	Methylprednisolone (2 mg/kg/day; intervention group) or dexamethasone (6 mg/day; control group)	47 patients in intervention group vs. 46 patients in control group hospitalised with COVID-19 pneumonia	Methylprednisolone results superior to dexamethasone
Saeed MAM, Mohamed AH, Owaynat AH. (2022) [38]	Comparison between methylprednisolone infusion and dexamethasone in COVID-19 ARDS mechanically ventilated patients	Prospective cohort study	Methylprednisolone 2 mg/kg/day IV vs. 192 dexamethasone 6 mg/day	222 patients treated with MP vs. 192 patients treated with dexamethasone admitted in ICU with confirmed diagnosis of SARS-CoV-2 pneumonia	Inflammatory markers for cytokine storm were improved in the methylprednisolone group in comparison to the patients treated with dexamethasone
Salton F, Confalonieri P, Centanni S, Mondoni M, Petrosillo N, Bonfanti P, et al. (2022) [39]	Prolonged higher dose methylprednisolone vs. conventional dexamethasone in COVID-19 pneumonia: A randomised controlled trial (MEDEAS)	Multicentre, open-label randomised clinical trial	Methylprednisolone 80 mg IV in continuous daily infusion for 8 days followed by slow tapering vs. dexamethasone 6 mg daily	337 patients treated with methylprednisolone vs. 340 in dexamethasone group with COVID-19 pneumonia requiring oxygen or non-invasive respiratory support	No significant differences in mortality between the two groups
Tyrosine Kinase Inhibitors					
Ely EW, Ramanan AV, Kartman CE, de Bono S, Liao R, Piruzeli MLB, et al. (2022) [44]	Efficacy and safety of baricitinib plus standard of care for the treatment of critically ill hospitalised adults with COVID-19 on invasive mechanical ventilation or extracorporeal membrane oxygenation: An exploratory, randomised, placebo-controlled trial	Multinational, phase 3, randomised, double-blind, placebo-controlled trial	Baricitinib 4 mg vs. placebo combination with standard of care	51 patients treated with baricitinib vs. 50 in placebo group hospitalised with laboratory-confirmed SARS-CoV-2 infection, mechanically ventilated or in extracorporeal membrane oxygenation	Treatment with baricitinib significantly reduced 28-day all-cause mortality compared with placebo
Kalil AC, Patterson TF, Mehta AK, Tomashek KM, Wolfe CR, Ghazaryan V, et al. (2021) [45]	Baricitinib plus remdesivir for hospitalized adults with COVID-19	Double-blind, randomised, placebo-controlled trial	Remdesivir plus baricitinib 4 mg daily or remdesivir alone (control)	515 patients receiving combination treatment vs. 518 in control group in in hospitalised adults with COVID-19	Baricitinib plus remdesivir was superior to remdesivir alone in reducing recovery time and accelerating improvement in clinical status
Bronte V, Ugel S, Tinazzi E, Vella A, De Sanctis F, Canè S, et al. (2020) [46]	Baricitinib restrains the immune dysregulation in patients with severe COVID-19	Observational longitudinal trial	Baricitinib 4 mg twice daily for 2 days, then 4 mg daily for 7 days	20 patients treated with baricitinib plus standard care vs. 56 patients treated only with standard care	Baricitinib prevented the progression to a severe form of SARS-CoV-2 pneumonia
Abani O, Abbas A, Abbas F, Abbas J, Abbas K, Abbas M, et al. (2022) [47]	Baricitinib in patients admitted to hospital with COVID-19 (RECOVERY): A randomised, controlled, open-label, platform trial and updated meta-analysis	Randomised, controlled, open-label, platform trial	Baricitinib 4 mg/daily with usual of care vs. standard of care alone	514 patients receiving baricitinib vs. 546 in standard of care alone group of hospitalised patients with COVID-19	13% proportional reduction in mortality in baricitinib group
Cao Y, Wei J, Zou L, Jiang T, Wang G, Chen L, et al. (2020) [48]	Ruxolitinib in treatment of severe coronavirus disease 2019 (COVID-19): A multicenter, single-blind, randomized controlled trial	Prospective, multicentre, single-blind, randomised controlled phase II trial	Ruxolitinib plus standard-of-care treatment vs. placebo based on standard of care treatment	22 patients receiving ruxolitinib vs. 21 patients treated with standard of care with severe COVID-19 pneumonia	No statistical difference between the two groups
Han MK, Antila M, Ficker JH, Gordeev I, Guerreros A, Bernus AL, et al. (2022) [49]	Ruxolitinib in addition to standard of care for the treatment of patients admitted to hospital with COVID-19 (RUXCOVID): A randomised, double-blind, placebo-controlled, phase 3 trial	International, randomised, double-blind, phase 3 trial	Ruxolitinib 5 mg twice daily or placebo plus standard of care	287 patients treated with ruxolitinib plus standard care vs. 145 patients with only standard of care in patients hospitalised, but not on mechanical ventilation or in the ICU	No benefit in the group treated with ruxolitinib
Abani O, Abbas A, Abbas F, Abbas M, Abbasi S, Abbass H, et al. (2021) [55]	Tocilizumab in patients admitted to hospital with COVID-19 (RECOVERY): A randomised, controlled, open-label, platform trial	Randomised, controlled, open-label, platform trial	Tocilizumab vs. usual care	621 patients allocated tocilizumab and 729 patients treated with usual care hospitalised with COVID-19	Tocilizumab improved survival and other clinical outcomes
Rosas IO, Bräu N, Waters M, Go RC, Hunter BD, Bhagani S, et al. (2021) [56]	Tocilizumab in hospitalized patients with severe COVID-19 pneumonia	Phase 3, international, randomised, double-blind, placebo-controlled trial	Single IV infusion of tocilizumab (8 mg/kg) vs. placebo	94 patients in the tocilizumab group vs. 144 in the placebo group with severe COVID-19 pneumonia	Tocilizumab did not significantly improve clinical status or lower mortality than placebo at 28 days
Salama C, Han J, Yau L, Reiss WG, Kramer B, Neidhart JD, et al. (2021) [57]	Tocilizumab in patients hospitalized with COVID-19 pneumonia	Randomised, double-blind, placebo-controlled, phase 3 trial	One or two IV infusion of tocilizumab (8 mg/kg) vs. placebo	249 patients in the tocilizumab group and 128 patients in the placebo group with COVID-19 pneumonia not receiving MV	Tocilizumab reduced the likelihood of progression to mechanical ventilation or death, did not improve survival
Somers EC, Eschenauer GA, Troost JP, Golob JL, Gandhi TN, Wang L, et al. (2021) [58]	Tocilizumab for treatment of mechanically ventilated patients with COVID-19	Single-centre cohort study	Single IV infusion of tocilizumab (8 mg/kg) vs. placebo	78 patients in tocilizumab vs. 76 in placebo group: tocilizumab needing MV	Tocilizumab treatment resulted in lower mortality despite higher superinfection occurrence
The REMAP-CAP Investigators (2021) [59]	Interleukin-6 receptor antagonists in critically ill patients with COVID-19	International, adaptive platform trial	Tocilizumab (8 mg/kg) vs. sarilumab 400 mg vs. standard care (control)	353 patients in tocilizumab, 48 in sarilumab, and 402 in control group in ICU critically ill patients	Tocilizumab and sarilumab both improved outcomes, including survival in the considered cohort of patients
Della-Torre E, Campochiaro C, Cavalli G, De Luca G, Napolitano A, La Marca S, et al. (2020) [60]	Interleukin-6 blockade with sarilumab in severe COVID-19 pneumonia with systemic hyperinflammation: An open-label cohort study	Open-label observational study	Sarilumab 400 mg plus standard of care vs. standard care alone	28 patients in sarilumab group vs. 28 patients in control group with in severe COVID-19 pneumonia and hyperinflammation (elevated inflammatory markers and serum IL-6 levels)	No significant difference in mortality between the two groups at day 28
Lescure F-X, Honda H, Fowler RA, Lazar JS, Shi G, Wung P, et al. (2021) [61]	Sarilumab in patients admitted to hospital with severe or critical COVID-19: A randomised, double-blind, placebo-controlled, phase 3 trial	Randomised, double-blind, placebo-controlled, multinational phase 3 trial	Sarilumab 400 mg vs. Sarilumab 200 mg vs. placebo	84 patients receiving placebo vs. 159 in sarilumab 200 mg vs. 173 sarilumab 400 mg in hospitalised patients with COVID-19 requiring supplemental oxygen	No efficacy in patients treated with sarilumab
Kyriazopoulou E, Poulakou G, Milionis H, Metallidis S, Adamis G, Tsiakos K, et al. (2021) [62]	Early treatment of COVID-19 with anakinra guided by soluble urokinase plasminogen receptor plasma levels: A double-blind, randomized controlled phase 3 trial	Pivotal, confirmatory, phase 3, double-blind randomised controlled trial	Anakinra vs. placebo	189 placebo vs. 405 anakinra in patients with rapidly deteriorating respiratory function	Reduction of 28-day mortality and hospital stay
Huet T, Beaussier H, Voisin O, Jouveshomme S, Dauriat G, Lazareth I, et al. (2020) [63]	Anakinra for severe forms of COVID-19: A cohort study	Retrospective cohort study	Anakinra subcutaneous plus standard care vs. standard care alone	52 patients in anakinra group vs. 44 patients in control group with severe bilateral COVID-19 pneumonia	Reduction in need for invasive mechanical ventilation in ICU and mortality
Kyriazopoulou E, Huet T, Cavalli G, Gori A, Kyprianou M, Pickkers P, et al. (2021) [64]	Effect of anakinra on mortality in patients with COVID-19: A systematic review and patient-level meta-analysis	Systematic review and individual patient-level meta-analysis	Anakinra with standard of care vs. placebo vs. both	1185 patients with SARS-CoV-2 infection	Anakinra reduced the mortality risk in patients admitted to hospital with moderate to severe COVID-19 pneumonia
Anti-complement therapies: anti-C5a					
Vlaar APJ, de Bruin S, Busch M, Timmermans SAMEG, van Zeggeren IE, Koning R, et al. (2020) [69]	Anti-C5a antibody IFX-1 (vilobelimab) treatment versus best supportive care for patients with severe COVID-19 (PANAMO): An exploratory, open-label, phase 2 randomised controlled trial	Exploratory, open-label, randomised phase 2 trial	Vilobelimab with best supportive care vs. best supportive care alone	15 patients in vilobelimab group vs. 15 patients in control group with severe COVID-19 pneumonia	Assessed safety of vilobelimab
Vlaar APJ, Witzenrath M, van Paassen P, Heunks LMA, Mourvillier B, de Bruin S, et al. (2022) [70]	Anti-C5a antibody (vilobelimab) therapy for critically ill, invasively mechanically ventilated patients with COVID-19 (PANAMO): A multicentre, double-blind, randomised, placebo-controlled, phase 3 trial.	Randomised, double-blind, placebo-controlled, multicentre phase 3 trial	Vilobelimab 800 mg IV for max. 6 days with best supportive care vs. best supportive care alone	54 patients in the vilobelimab group and 77 patients in control group with SARS-CoV-2 infection receiving invasive mechanical ventilation	Vilobelimab ameliorated survival in invasive ventilated patients and decreased mortality
Interferon					
Monk PD, Marsden RJ, Tear VJ, Brookes J, Batten TN, Mankowski M, et al. (2021) [71]	Safety and efficacy of inhaled nebulised interferon beta-1a (SNG001) for treatment of SARS-CoV-2 infection: A randomised, double-blind, placebo-controlled, phase 2 trial	Randomised, double-blind, placebo-controlled, phase 2 pilot trial	Inhaled interferon beta-1a vs. placebo	48 patients received inhaled interferon and 50 received placebo hospitalised with confirmed COVID-19 infection	Greater odds of improvement and rapid recovery in interferon group
Kalil AC, Mehta AK, Patterson TF, Erdmann N, Gomez CA, Jain MK, et al. (2021) [72]	Efficacy of interferon beta-1a plus remdesivir compared with remdesivir alone in hospitalised adults with COVID-19: A double-blind, randomised, placebo-controlled, phase 3 trial	Double-blind, randomised, placebo-controlled trial	Remdesivir IV and interferon beta-1a or remdesivir	487 in interferon plus remdesivir group vs. 482 remdesivir group in hospitalised adults with SARS-CoV-2 infection	Adding interferon showed no significant improvement
Anti-GM-CSF					
Temesgen Z, Assi M, Shweta FNU, Vergidis P, Rizza SA, Bauer PR, et al. (2020) [77]	GM-CSF neutralization with lenzilumab in severe COVID-19 pneumonia: A case cohort study	Case cohort study	Lenzilumab 600 mg	12 patients treated with lenzilumab vs. 27 untreated patients with COVID-19 pneumonia and risk factors for poor outcomes	Assessment of safety of lenzilumab and faster improvement

## 8. Current Guidelines

Current WHO guidelines strongly recommend, for severe and critical forms of COVID-19, the use of corticosteroids together with an IL-6 receptor blocker and baricitinib. In addition, the use of ruxolitinib or tofacitinib is suggested only in the case of unavailability of baricitinib or other IL-6 receptors [4]. 

Currently, the US Food and Drug Administration (FDA) has only approved baricitinib and tocilizumab for patients with severe SARS-CoV-2 pneumonia who require supportive oxygen, non-invasive or invasive mechanical ventilation, or extracorporeal membrane oxygenation (ECMO), as listed in Table 2. Anakinra is licensed for treatment with ruxolitinib or tofacitinib. Anakinra is licensed for the treatment of COVID-19 in hospitalised adults with pneumonia who require supplemental oxygen and who are at risk of progressing to severe respiratory failure and are likely to have elevated plasma-soluble urokinase plasminogen activator receptor (suPAR), according to the FDA’s Emergency Use Authorization [50].

Currently, in the European Union, the European Medicines Agency (EMA) has only approved tocilizumab for intravenous infusion for patients with severe COVID-19 already on corticosteroid therapy. The EMA has also approved anakinra with the same indications as the FDA, specifically requiring blood levels of suPAR of at least 6 ng/mL [51].

## 9. Discussion

In addition to supportive therapy, glucocorticoids have become the mainstay and standard of care for severe COVID-19. In particular, current guidelines confirm the evidence that GC administration reduces mortality, with effectively manageable side effects in clinical practice, mostly related to the development of hyperglycaemia or hypernatremia. The use of corticosteroids is also particularly manageable in clinical practice compared to the other agents proposed for the treatment of SARS-CoV-2 infections, given physicians’ greater experience with these types of drugs. Currently, the guidelines do not recommend a single molecule; treatment can be administered orally or systemically (taking into account patient characteristics), treatment duration varies from at least 5 to 14 days, and dosing depends on the type of molecule used [4,15,43,79]. Further studies should focus on which steroid treatment protocol is the most appropriate and which molecules are best suited to control the enormous inflammatory response that characterises the cytokine storm. 

One possible area of investigation could be the efficacy of combination therapy with monoclonal antibodies and steroid therapy. In addition, an update of the European Respiratory Society (ERS) guidelines, published in June 2022, and the current WHO guidelines, updated in January 2023, strongly recommend the use of monoclonal antibodies against IL-6 and JAK inhibitors together with GCs. In contrast, conditional recommendations have been made for the use of IFN-β and of IL-1 receptor antagonist monoclonal antibody [4,43]. 

The current results already suggest the option of treating the patient according to the disease phenotype, considering the severity of COVID-19 pneumonia at onset, the inflammatory status detectable by measurement of serum inflammation indices, and the level of respiratory support required (e.g., oxygen only, non-invasive or invasive ventilation). Future research will also likely lead to the identification of genomic or transcriptomic patterns that could allow a deeper understanding of the disease and could have both prognostic and therapeutic relevance. 

The discovery of these mechanisms could also clarify why, in the pre-vaccine era, the spectrum of disease presentation was so differentiated in patients with similar baseline characteristics. 

Currently, several classes and treatment regimens are available for the treatment of patients with severe COVID-19. Several studies, including large numbers, have demonstrated the greater or lesser efficacy of these drugs and have allowed the development of guidelines for their use based on host characteristics. What is critical to understand today is the correct timing of the initiation of these therapies to ensure their efficacy. It will therefore be essential to identify the biological markers that can guide the clinician not only in choosing the appropriate therapy, but also in the correct therapeutic timing. This evidence and future discoveries bring us closer to tailored therapy based on the clinical features, severity of the disease, and its biological and genetic profile.

## Figures and Tables

**Table 2:** Actual indications for severe SARS-CoV-2 pneumonia approved by EMA and FDA.

Therapy	FDA Indications	EMA Indications
Tocilizumab	Hospitalised adults receiving systemic corticosteroids and requiring oxygen, NIV, IVM, or ECMO	Hospitalised adults receiving corticosteroid via OS or IV and requiring oxygen, NIV, or IVM
Baricitinib	Hospitalised adults receiving systemic corticosteroids and requiring oxygen, NIV, IV, or ECMO	Not approved in Europe
Anakinra	Adults with pneumonia requiring supplemental oxygen at risk of progressing to severe respiratory failure and likely to have elevated plasma suPAR *	Adults with pneumonia requiring supplemental oxygen, at risk of developing severe respiratory failure with suPAR blood level of at least 6 ng/mL

* FDA’s Emergency Use Authorization; NIV: non-invasive mechanical ventilation, IVM: invasive mechanical ventilation; ECMO: extracorporeal membrane oxygenation; OS: oral; IV: intravenous; suPAR: soluble urokinase plasminogen activator receptor.

## Data Availability

Data sharing not applicable.
